# Translation, Cultural Adaptation, and Psychometric Validation of the European Portuguese Version of the Iceland-Expressive Family Functioning Questionnaire (ICE-EFFQ)

**DOI:** 10.1177/10748407231205038

**Published:** 2023-12-01

**Authors:** Maria do Carmo Lemos Vieira Gouveia, Eydis Kristin Sveinbjarnardottir, Maria João Barreira Rodrigues, Rita Maria Lemos Baptista Silva, Márcia Sílvia Baptista, Maria Adriana Pereira Henriques

**Affiliations:** 1 Nursing Research, Innovation and Development Centre of Lisbon (CIDNUR), Nursing School of Lisbon, Portugal; 2 University of Madeira, Funchal, Portugal; 3 University of Iceland, Reykjavik, Iceland; 4 University of Akureyri, Iceland; 5 Regional Government of Madeira, Funchal, Portugal; 6 University of Lisbon, Portugal

**Keywords:** depression, expressive family functioning, Iceland-Expressive Family Functioning Questionnaire, psychometric testing

## Abstract

A family’s experience of mental illness can change the family’s functioning. In clinical contexts, valid and reliable instruments that assess family functioning, therapeutic changes, and the effects of family nursing interventions are needed. This study focuses on the linguistic and cultural adaptation of the Iceland-Expressive Family Functioning Questionnaire (ICE-EFFQ) to European Portuguese and examines the psychometric properties of this instrument. A non-random sample of 121 Portuguese depressed patients and their relatives completed the questionnaire. Principal components analysis extracted 4 factors, explaining 55.58% of the total variance. Confirmatory factor analysis revealed acceptable adjustment quality indices. Cronbach’s alpha coefficient was adequate for the global scale *α* = .86 and for the 4 subscales: communication *α* = .79, expression of emotions *α* = .68, problem-solving *α* = .71, and cooperation *α* = .61. The Portuguese version of ICE-EFFQ is a sensitive, valid, and reliable instrument for use with Portuguese families with adult members with depression and can be valuable in assessing these families’ expressive functioning, before and after intervention.

Mental illness is a family affair (Price et al., 2021; Wright & Bell, 2021; Wright & Leahey, 2013) which may cause changes in family functioning (MacFarlane, 2003; Marshall & Harper-Jaques, 2008; Sell et al., 2021; Sveinbjarnardottir & Svavarsdottir, 2019; Yorganson & Stott, 2017). The illness process affects both instrument and expressive functioning in families (Hill et al., 2022; Kassem et al., 2022; Wright & Leahey, 2013; Yorganson & Stott, 2017).

Depression has been shown to have a significant effect on family functioning, that is, with communication, affective involvement, problem-solving, and moreover with overall family functioning (J. de Souza et al., 2011; Dibenedetti et al., 2012; Park & Jung, 2019; Sveinbjarnardottir & Svavarsdottir, 2019).

Family functioning is a concept that encompasses the dynamics and relationships within a family system (Sell et al., 2021; Skinner et al., 2000) and is focused on the collective health of the family (Daches et al., 2018). According to the Calgary Family Assessment Model (CFAM) by Wright and Leahey (2013), family functioning includes both instrumental and expressive aspects. The instrumental aspects refer to daily life activities, such as dressing, eating, and hygiene, while the expressive aspects refer to communication, relationships, and problem-solving between family members (Wright & Leahey, 2013). These expressive aspects include emotional and verbal communication, power dynamics, beliefs, and connections.

Nurses must be educated to include families in the care for their ill family member (Chesla, 2010; Duhamel et al., 2015; Naef et al., 2021) and understand the importance of expressive functioning in evaluating family functioning (Wright & Leahey, 2013). The assessment of family functioning focuses on the patterns of interaction between family members and considers each member’s behavior in the context of the family system (Papadopoulos, 1995; Wright & Leahey, 2013). The family is viewed as a system of interacting members who influence and define each other within the family context.

Research has demonstrated the benefits of family interventions for both patients and family members (Chesla, 2010; Konradsen et al., 2018; Sveinbjarnardottir & Svavarsdottir, 2019). Family-centered interventions are crucial and should be offered to families, adults, and children, affected by mental illness, as they have been shown to improve family functioning (Beardslee et al., 2007; Sveinbjarnardottir & Svavarsdottir, 2019). Reliable and valid instruments for assessing expressive family functioning in families facing a mental illness, particularly depression, are important for detecting family needs, improving family functioning, and evaluating the effectiveness of nursing interventions.

In the assessment of family functioning, a variety of instruments have been used by health professionals (Åstedt-Kurki et al., 2002, 2009; Galán-González et al., 2021; Hohashi et al., 2008), including the Family Assessment Device (Epstein et al., 1978; Miller et al., 2000), the Family Functioning Health and Social Support questionnaire (Åstedt-Kurki et al., 2002, 2009), the Iceland-Expressive Family Functioning Questionnaire (ICE-EFFQ) (Konradsen et al., 2018; Sveinbjarnardottir et al., 2012), and the Feetham Family Functioning Scale (Hohashi et al., 2008; Roberts & Feetham, 1982). The ICE-EFFQ (Sveinbjarnardottir et al., 2012) is a highly regarded instrument that measures expressive family functioning and has been shown to be useful, valid, and reliable in various clinical settings, with families facing acute and chronic illnesses (Dieperink et al., 2018; Konradsen et al., 2018; Sveinbjarnardottir et al., 2012). The ICE-EFFQ (Sveinbjarnardottir et al., 2012) has been tested and successfully used to assess family functioning in families with acute and chronic illnesses (Galán-González et al., 2021; Kamban & Svavarsdottir, 2013; Svavarsdottir et al., 2012), acute psychiatric patients (Sveinbjarnardottir et al., 2013), and those with oncological disease (Dieperink et al., 2018; Konradsen et al., 2018; Svavarsdottir & Sigurdardottir, 2013).

The study aimed to adapt the ICE-EFFQ (Sveinbjarnardottir et al., 2012) to the Portuguese language and culture, and to evaluate its psychometric properties. The goal was to make the adapted questionnaire available for use by health care professionals and researchers in Portuguese families dealing with acute or chronic mental illness in a family member. The decision to study families affected by acute or chronic depression was influenced by three main factors. First, mental health professionals in the Autonomous Region of Madeira identified them as a priority focus. Second, Portuguese epidemiological data showed a high prevalence of mental illness and mood disorders (de Almeida, 2018; de Almeida et al., 2013), with depressive disorders presenting higher levels of severity compared with other groups of psychiatric pathologies (de Almeida, 2018). Third, research has shown that depression affects the behavior, emotions, communication, and well-being of individuals and their families (Källquist & Salzmann-Erikson, 2019; Sveinbjarnardottir & Svavarsdottir, 2019; World Health Organization, 2019) and is associated with impaired family functioning (Daches et al., 2018; Pérez et al., 2018; Sell et al., 2021; Wang & Zhao, 2013). These factors point to the importance of addressing the needs of families affected by depression in mental health care.

Given the fact that, in Portugal, there are no known instruments to measure family expressive functioning in families facing an acute or chronic mental illness of a relative, and that valid and reliable instruments able to measure the therapeutic change and the effectiveness of family interventions are greatly needed (Chesla, 2010; Galán-González et al., 2021; Sveinbjarnardottir et al., 2012), we decided to translate the ICE-EFFQ (Sveinbjarnardottir et al., 2012) to European Portuguese and to test its psychometric properties.

## Purpose of This Study

The purpose of this study was to develop a linguistic and cultural adaptation of the ICE-EFFQ (Sveinbjarnardottir et al., 2012) to European Portuguese and to assess its psychometric properties for future application by health professionals and researchers in Portuguese families facing acute or chronic mental illness of their members.

## Method

A descriptive cross-sectional study was conducted in two phases. Phase 1 involved the cultural and linguistic adaptation of the ICE-EFFQ to European Portuguese comprising a cycle of translation and back-translation, followed by the linguistic screening and cultural adaptation, performed by experts’ analysis and a cultural pre-test with 10 patients and family members from the target population. Phase 2 involved the psychometric testing of the Portuguese version of the instrument to perform principal components analysis, confirmatory factor analysis, and reliability assessment.

### The Iceland-Expressive Family Functioning Questionnaire (ICE-EFFQ)

The ICE-EFFQ (Sveinbjarnardottir et al., 2012) is a self-report questionnaire developed and psychometrically assessed in three different studies by a group of Icelandic nurses who are experts in family nursing. The ICE-EFFQ measures the concept of expressive functioning in families that are dealing with the acute or chronic illness of their members and defines these families’ expressive functioning as a multidimensional concept that covers the expression of emotions, collaboration and problem-solving, communication, and behavior (Sveinbjarnardottir et al., 2012). It consists of 17 items and 4 factors, scored on a Likert-type scale ranging between 1 (almost never) and 5 (almost always). The ICE-EFFQ is based on the functional assessment category of the CFAM developed by Wright and Leahey (2013), which reflects the response of families to acute or chronic illness of their members (Sveinbjarnardottir et al., 2012; Wright & Leahey, 2013). It was found to be valid, reliable, and to have good internal consistency, with adequate alpha values for Cronbach’s coefficient for the total scale α = .922 and for all subscales: expressing emotions *α* = .737; collaboration and problem-solving *α* = .809; communication *α* = .829; and behavior *α* = .813 (Sveinbjarnardottir et al., 2012).

### Phase I—Linguistic and Cultural Adaptation of the Instrument

**Figure 1.**
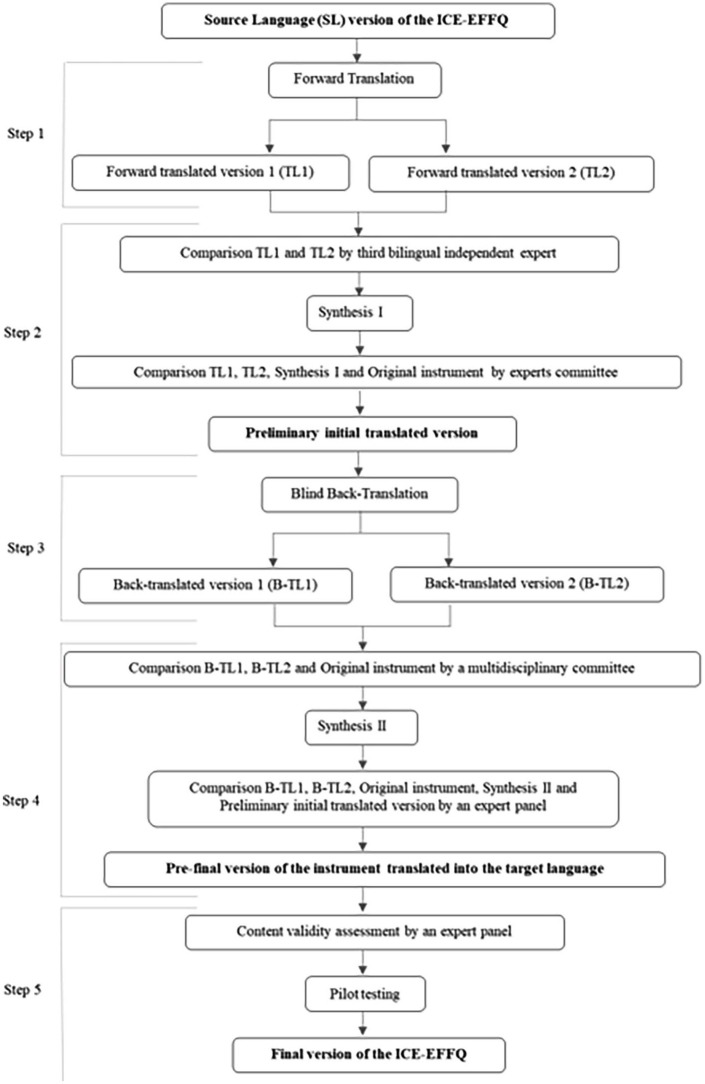
Translation and cross-cultural adaptation process of ICE-EFFQ into European Portuguese. *Note.* ICE-EFFQ = Iceland-Expressive Family Functioning Questionnaire.

In the translation and cultural adaptation of the ICE-EFFQ into European Portuguese, the guidelines proposed by Sousa and Rojjanasrirat (2011) were adopted. The process was developed in five steps (see Figure 1).

#### Step 1. Forward Translation of the Original Instrument Into European Portuguese

The instrument’s adaptation into European Portuguese started with the linguistic component, through a cycle of translation and back-translation (Sousa & Rojjanasrirat, 2011). The original instrument in English was translated into Portuguese by two bilingual experts who are independent, certified, native Portuguese speakers with distinct backgrounds. The first expert was familiar with the terminology used in the field of health and the instrument construct’s contents in Portuguese. The second expert was familiar with the cultural and linguistic characteristics of the population and the Portuguese language, although having no knowledge of medical terminology or the instrument construct. Two provisional translations of the original instrument were produced, simultaneously covering medical language and the language usually spoken in the target language, considering their cultural characteristics.

#### Step 2. Comparison of the Two Translated Versions of the Instrument: Synthesis I

Upon receipt of the two translations, a third-party, bilingual, independent expert was brought in who is a native Portuguese speaker and has good knowledge of the English language and of the instrument construct’s contents, in both Portuguese and English. This expert then compared the instructions, items, and response format in the two translated versions, with one another and with the original instrument, in relation to ambiguities and discrepancies in words, sentences, and meaning, and developed a synthesis of the translated versions (synthesis I). Then a first meeting of experts was held, with the participation of the main research team (MRT) and the three bilingual experts, who analyzed the main differences between the two translated versions, the synthesis I and the original instrument. In addition, questions and differences related to semantics, concepts, and cultural aspects were discussed, and, by consensus, the preliminary initial translated version of the instrument into Portuguese was produced.

#### Step 3. Blind Back-Translation of the Preliminary Initial Translated Version of the Instrument Into English

The questionnaire was subsequently back-translated into the original language by two bilingual, independent, certified, experts, who are native English speakers with the same characteristics as the experts of step 1. None of the experts had prior knowledge of the instrument to be back-translated. Based on the preliminary initial translated version of the instrument into Portuguese, two independent back-translated versions in the original language were produced by native English-speaking experts.

#### Step 4. Comparison of the Two Back-Translated Versions of the Instrument: Synthesis II

Next, a multidisciplinary committee consisting of the MRT, and all bilingual and bicultural translators involved in the previous steps, compared each one of the two back-translations and the original instrument with respect to the similarity of the instructions, items, and response format, wording, phrasal structure, similarity of meaning and relevance of sentences. All ambiguities and discrepancies regarding cultural meaning and idioms in words and sentences were discussed in the committee and decided by consensus. A synthesis of the back-translated versions was then produced (synthesis II) and sent along with both back-translations to the first author of the original instrument, who provided insights on the construct of the instrument and clarified the meaning of some words and expressions. Minor linguistic adjustments to the preliminary initial translation of the instrument into Portuguese and synthesis II in English were made.

Two meetings followed with a panel of three experts in family nursing, mental and psychiatric health nursing, and community nursing, with the dual function of assessing, reviewing, and consolidating the instructions, items, and answer format of the two back-translations and synthesis II, with conceptual, semantic, and content equivalence, and developing the pre-final version of the instrument in the target language, for pilot testing and psychometric assessment. The expert panel carefully compared the two back-translations one another and with the original version, the synthesis II, and the preliminary initial translated version into Portuguese, regarding format text, phrasal and grammatical structure, colloquial parlance, language, similarity of meanings, cultural significance, and relevance. Small words were changed to ensure cultural and conceptual equivalence, and all ambiguities and discrepancies were discussed and resolved by consensus. The expert panel assessed the conceptual equivalence of the instructions, items, and the response format, by completing a dichotomous scale (clear/unclear) that obtained 100% agreement among the evaluators. This process resulted in the pre-final version of the instrument in Portuguese, which was called the “*Questionário do Funcionamento Expressivo da Família* (QFEF)” (“Questionnaire on the Expressive Family Functioning (QEFF)”).

#### Step 5. Content Validity Assessment and Pilot Testing of the Pre-Final Version of the Instrument in Portuguese

To analyze the content validity of the pre-final version of the instrument in Portuguese (QFEF), a panel of three family nursing experts with experience in academic and clinical practice was selected. To assess the relevance of each item for the underlying dimensions that the QFEF intends to measure, the 3 experts completed a content validity index (CVI) using a 4-point Likert-type scale, scored from 1—Non relevant to 4—Very relevant and succinct (Polit & Beck, 2006, 2017). The content validity of the instrument was estimated by assessing the content validity at the item level (I-CVI) and at the scale level (S-CVI). The values for I-CVI and S-CVI should not be less than 1.0 when there are fewer than 5 expert evaluators (Polit & Beck, 2006, 2017; Streiner et al., 2015). The content validity of the scale, calculated by the assumed mean method, measured homogeneous results whose values evidence strong relevance of the items in the Portuguese version of the ICE-EFFQ: mean I-CVI = 1.0; S-CVI/UA = 1.0; and S-CVI/Ave = 1.0.

A cultural pre-test was performed with 10 participants taken from the target population, to strengthen the conceptual, semantic, and content equivalency of the translated instrument, to improve the phrasal structure of the instructions, items, and response format, and to allow for easy understanding by the target population (Polit & Beck, 2004, 2017) Each participant was invited to evaluate the clarity of the instrument’s instructions, items, and response format on a dichotomous scale (clear/unclear) and to offer suggestions about how to rewrite the statements they thought were unclear (Sousa & Rojjanasrirat, 2011). A 100% agreement was obtained between the evaluators in the sample, for the clarity of the instructions and items, and 90% for the clarity of the response format. Notably, 10% of the participants suggested changing the ascending order of the “generally” and “almost always” answers, to “almost always” and “generally,” or replacing the “almost always” option with “always.” The committee decided to keep the response format of the original authors, so there were no modifications in the instructions, items, or response format, after the application of the pre-test. This step aimed to review and refine the items from the pre-final version of the instrument, and generate the final psychometric instrument, with adequate estimates for reliability, homogeneity, and validity, and with a stable factor structure, and/or model adjustment (Sousa & Rojjanasrirat, 2011). As a result of this step, the final European Portuguese version of the ICE-EFFQ was achieved.

### Phase II—Psychometric Testing

This was followed by a complete psychometric testing in a sample taken from the target population (Streiner et al., 2015).

#### Sample and Participants

The target population of the study consisted of Portuguese families with adult members with depression, living in the Autonomous Region of Madeira (RAM). The participants were recruited in the health centers and psychiatric inpatient facilities of the RAM after depressed patients were identified by mental health specialist nurses, general care nurses, and family doctors. The sample included depressed patients and their family members. Recruitment and data collection took place from May 2015 to February 2017. The inclusion criteria were as follows: Patients aged between 18 and 75 years old; diagnosed with depression, according to the International Statistical Classification of Diseases and Related Health Problems, 10th Revision (ICD-10), *Diagnostic and Statistical Manual of Mental Disorders* (4th ed.; *DSM-IV*; American Psychiatric Association, 1994), and/or International Classification of Primary Care, 2nd edition (ICPC-2), with a score >20 on the “Inventário de Avaliação Clínica da Depressão-IACLIDE” [Clinical Assessment Inventory of Depression] (Serra, 1994), or <20, if there is a history of depressive symptomatology and medical diagnosis of depressive disorder (according to the ICD-10, *DSM-IV*, and/or ICPC-2) within a year before the assessment. Family members aged 18 years or older, with or without blood ties to the depressed person, who are referred to by the member with depression as family, and designated by him to take part in the study. The exclusion criteria were as follows: depression secondary to another clinical condition; clinical history of schizophrenia or bipolar disorder; and active psychotic and/or delusional symptoms at the time of assessment.

The data were collected by nurses specializing in mental and psychiatric health and by the investigator, and a non-random sample of 121 participants (7.12 per item of the scale) was formed, including 55 families with recent experience of depression. This was defined as a diagnosis of acute or chronic depression of 1 adult family member in the period of 1 year before the time of assessment (Sveinbjarnardottir et al., 2012). The sample size, as a rule, should consider a certain number of subjects for each item of the scale; an acceptable subject/item ratio of at least between 5 and 10 participants per item is suggested (Marôco, 2021a; Nunnally & Bernstein, 1994; Polit & Beck, 2017; Streiner et al., 2015). The average completion time of the Portuguese version of the ICE-EFFQ was 10 minutes, with a standard deviation of 7.5 minutes and minimum and maximum completion times of 3 and 57 minutes, respectively, and 75% of the respondents took 11.5 minutes to complete the questionnaire. Additional structured questions to provide information on sociodemographic and health variables (gender, age, marital status, education, family relationships, work situation, and psychiatric health status of the participants) were filled out by the nurses through a data collection interview at the nursing consultation.

#### Ethical Considerations

The study was developed according to the international ethical principles of scientific research embodied in the Helsinki Declaration (World Medical Association, 2013, 2018). Permission was requested and granted via e-mail from the authors of the ICE-EFFQ for the translation, cultural adaptation, and psychometric validation of the instrument in European Portuguese. The study was approved by the Ethics Committee of the Regional Health Service of RAM (Nº 51/2014). All adult participants diagnosed with depression gave their prior consent for the inclusion of their families in the study and designated family members for contact and recruitment. The participants were informed about the study’s objectives, purpose, and implications, about the confidentiality agreement and the anonymity granted by the ethical principles of research, and about the right to participate voluntarily and to withdraw at any time and without any consequences, should they wish to do so. All participants received a document containing information about the research subject and purpose and signed an informed consent form. The investigator’s telephone number and e-mail were made available to the participants, to clarify any doubts during the investigation process.

#### Construct Validity

To measure the construct validity of the Portuguese version of the ICE-EFFQ, a complete psychometric test of the final version of the translated instrument was conducted. The instrument’s metric properties were assessed through validity studies, involving exploratory factor analysis (EFA) by the principal component method, to determine dimensionality, and confirmatory factor analysis (CFA), to confirm the factor structure (Marôco, 2021a, 2021b; Pestana & Gageiro, 2014; Sousa & Rojjanasrirat, 2011).

##### Exploratory Factor Analysis (EFA)

In EFA, the principal component analysis method was used to verify that the variables of the Portuguese version synthesized the same factors as the original version of the ICE-EFFQ, and by use of varimax orthogonal rotation, to determine the weight or loading of each item in the extracted factor (Marôco, 2021b; Pestana & Gageiro, 2014). To assess the instrument’s adequacy to proceed to factor analysis, we used the Kaiser–Meyer–Olkin (KMO) test for sampling adequacy, and for the factorability of the correlation matrices, Bartlett’s test of Sphericity of χ^2^ (Marôco, 2021b; Pestana & Gageiro, 2014). Factor analysis is considered to show that an instrument is adequate for the variables when the value for KMO is between .80 and .90, and very adequate when this coefficient presents higher values (Kaiser, 1974; Marôco, 2021b; Pestana & Gageiro, 2014), and the Bartlett sphericity test yields *p* < .001. As a criterion for factor retention, the cutoff point, or saturation of items in each factor, was set at ≥.40, with eigenvalues greater than 1 (Kaiser criterion), total variance explained by the factors and the instrument’s total, and the “scree plot,” or slope chart, proposed by Cattell (Marôco, 2021b; Pestana & Gageiro, 2014). Mean, standard deviation, and communalities (*h*^2^) were assessed. In the extraction of factors, the underlying theoretical perspective and the results of the factor analysis were considered, with different factor structures tested.

##### Confirmatory Factor Analysis (CFA)

For CFA,a covariance matrix was used, and maximum likelihood estimation was adopted for parameter estimation (Marôco, 2021a, 2021b; Pestana & Gageiro, 2014; Polit & Beck, 2017). The following statistical procedures were considered: (a) item sensitivity evaluated by asymmetry (*Sk* ≤ 3) and flattening (*Ku* ≤ 7) (Marôco, 2021a); (b) quality of the global fit of the factorial model evaluated by using the following indices and reference values: Chi-square ratio versus degrees of freedom (*x*^2^*/df*). It is suggested to be less than 3 to be considered a good model fit (Marôco, 2021a; Soeken, 2010); goodness-of-fit index (GFI). Values greater than .90 are suitable, with GFI = 1 indicating a perfect fit (Marôco, 2021a, 2021b; Pestana & Gageiro, 2014; Soeken, 2010); comparative fit index (CFI). Values closer to 1 indicate better adjustment, and .90 is the reference to accept the model (Hu & Bentler, 1999; Marôco, 2021a); root mean square error of approximation (RMSEA) is a measure of the amount of error in the CFA (Marôco, 2021a). Values lower than .05 are indicative of a good fit between the proposed model and the observed matrix, although values lower than .08 are acceptable (Hu & Bentler, 1999; Marôco, 2021a; Pestana & Gageiro, 2014); root mean square residual (RMSR). The lower the RMSR (<.1), the better the adjustment, with RMSR = 0 indicating a perfect fit (Marôco, 2021a, 2021b); standardized root mean square residual (SRMR). A value of 0 indicates a perfect fit, values less than .10 are desired, and a value less than .08 is considered a good fit (Hu & Bentler, 1999; Soeken, 2010); (c) quality of the local fit of the factorial model assessed by factor weights (*λ* ≥ .50) and the individual reliability of items (*r*^2^ ≥ .25) (Marôco, 2021a); (d) composite reliability (CR), which estimates the internal consistency of the items relative to the factor, was assessed with the standardized Cronbach’s alpha for each of the factors. CR ≥ .70 indicates appropriate construct reliability, although, for exploratory investigations, lower values may be acceptable (Marôco, 2021a); (e) convergent validity analysis, obtained by average variance extracted (AVE), assesses how the items that reflect a factor strongly saturate this factor, that is, the behavior of these items is explained by this factor. Values of AVE ≥ .50 indicate adequate convergent validity (Marôco, 2021a); and (f) discriminant validity (DV) analysis, assessed by comparing the AVE for each factor with the squared Pearson correlation. There is evidence of DV when the squared correlation between the factors is lower than the AVE for each factor (Marôco, 2021a).

The model fit was based on the modification indices indicated by Analysis of Moment Structures (AMOS) “above 11; *p* < .001” and on theoretical considerations (Marôco, 2021a; Soeken, 2010).

#### Reliability Assessment

Reliability assessment, a measure that assures the data are stable or consistent, regardless of the context, the instrument, or the researcher (Polit & Beck, 2017; Ribeiro, 2010; Streiner et al., 2015), involved determination of the internal consistency and temporal stability: (a) internal consistency or homogeneity of items was conducted to assess the degree to which the items of the scale were measuring the same construct. It was estimated using the following: Cronbach’s alpha coefficient for each factor and the overall scale. A good internal consistency should exceed a .80 alpha (Cronbach, 1990; Pestana & Gageiro, 2014). The reference values were those recommended by Pestana and Gageiro (2014): >.9 very good; .8 to .9 good; .7 to .8 reasonable; .6 to .7 weak; <.6 unacceptable; Pearson’s correlation coefficient of the various items, assuming a global score as a reference value, with correlations >.20 (Marôco, 2021a). It seeks to determine the degree of item differentiation, in the same sense as the global test, since an item is more discriminative, the more discrepancy it provides between two groups (higher and lower values of the scale); (b) temporal stability, also understood as test–retest reliability, seeks to ascertain the stability of the instrument over time, that is, whether the instrument gives identical results when administered at different times. Test–retest reliability coefficients above .9 are considered high and between .7 and .8 are acceptable for research tools (Keszei et al., 2010; Streiner et al., 2015). To measure reliability, the questionnaire was administered to a subset of the sample (*n*=40), 3 weeks apart, according to the temporal spacing between measurements, recommended by the authors (A. C. de Souza et al., 2017; Keszei et al., 2010; Streiner et al., 2015). Test–retest correlation was assessed by calculating the Pearson correlation coefficient and the intraclass correlation coefficient (ICC). The ICC is one of the most used tests to estimate the stability of continuous variables, since it takes measurement errors, such as variations over time and systematic differences, into account (A. C. de Souza et al., 2017; Streiner et al., 2015; Streiner & Kottner, 2014).

## Data Analysis

The psychometric properties of the instrument were assessed, using the Statistical Package for Social Sciences (SPSS) Version 24 and the special module of SPSS AMOS Version 24. For the sociodemographic characterization of the sample, descriptive statistics were used, with measures of central tendency and dispersion, particularly absolute and relative frequencies, mean, median, minimum, maximum, standard deviation, and percentiles. In the application of statistical inference methods, namely, the Chi-square homogeneity test, a significance level of α = .05 was considered.

## Results

Participants were recruited in the health centers (66.1%) and psychiatric hospitals (33.9%) of RAM, by mental health specialist nurses (61.2%), general nurses (32.1%), and family doctors (6.6%). In Table 1, gender, age, civil status, education level, family relationship to the patient, and family type are summarized. There were 121 subjects, 67 family members (55.4%) mostly sons/daughters, spouses, and parents (85 %), and 54 patients, who completed the questionnaire. Most were female (66.1%), the mean age of the participants was 44.9 years old (*SD* = 14.5), and the majority (*n* = 71, 58.7%) were less than 50 years old (Male *n* = 24, 58.5%; Female *n* = 47, 58.8%). The difference between the proportions of subjects in the different age groups was not statistically significant (*p* value = 1.000>.05), which points to the homogeneity of the gender compared with the age group. Most participants (61.2%) had a lower level of education (Eurostat, 2022; Eurydice, 2022a, 2022b), 72.2% (*n*=88) belonged to the working population, and 24% (*n*=29) were unemployed. More than a half (*n*=68, 56.2%) had a stable source of income, while 20.7% (*n*=25) depended on social subsidies and 23.1% (*n*=28) had no source of income.

**Table 1.** Characteristics of the Participants (*N* = 121).

Participants variables	*N*	(%)
Gender		
Male	41	33.9
Female	80	66.1
Civil status		
Single	26	21.5
Married/living in a consensual union	74	61.2
Divorced/separated	13	10.7
Widower	8	6.6
Education level		
With no schooling	6	5.0
First to third cycle of schooling	74	61.2
Secondary school	19	15.7
High education	22	18.2
Total	121	100.0
Family relationship to the patient		
Patient	54	44.6
Family member		
Spouse	24	35.8
Father/mother	8	11.9
Son/daughter	25	37.3
Other relatives	10	14.9
Sub-total	67	100.0
Family type		
Nuclear	66	54.5
Single parent family	21	17.4
Reconstructed	5	4.1
Extended	25	20.7
Unitary	4	3.3
Total	121	100.0

Regarding current psychiatric diagnosis, most participants (*n* = 68, 56.2%) had a psychiatric disorder, 50.4% (*n* = 61) had depression, and less than a half had no psychiatric pathology, while 46.2% (*n* = 56) of the subjects showed a history of psychiatric pathology, 37.1% (*n*=45) had a history of depression, and almost 49% had no psychiatric antecedents (see Table 2). There were no missing values in the answers to the questionnaires, except in the descriptive data in the variables “Current psychiatric diagnosis” and “Psychiatric history,” where six family members gave no information.

**Table 2.** Current Psychiatric Diagnosis and Psychiatric History Distribution of Participants.

Current psychiatric diagnosis	No.	(%)
No psychiatric diagnosis	52	43.0
Depression	42	34.7
Depression with suicidal ideation and/or attempts	12	9.9
Depression and other psychiatric disorders	7	5.8
Other psychiatric disorders	7	5.8
Missing values	1	0.8
Total	121	100.0
Psychiatric history	No.	(%)
No psychiatric history	59	48.8
Depression	17	14.0
Depression with suicidal ideation and/or attempts	8	6.6
Depression and other psychiatric disorders	20	16.5
Other psychiatric disorders	11	9.1
Missing values	6	5.0
Total	121	100.0

### Construct Validity

#### Exploratory Factor Analysis (EFA)

The KMO test, a measure of sampling adequacy, was adequate (KMO = .834) to proceed to factor analysis (Marôco, 2021b; Pestana & Gageiro, 2014). The Bartlett sphericity test (*p* < .001) (χ^2^ = 620,824 [*df* = 136]; *p* < .001) also indicated that the matrix was suitable for analysis (Marôco, 2021b). The 17 items were then subjected to EFA utilizing the principal components method with varimax orthogonal rotation, with latent roots greater than 1, using values equal to or greater than .40 as a criterion for item saturation (Marôco, 2021b).

Table 3 shows item loadings, eigenvalues, variance accounted for each factor, and communalities. The final factor solution allowed the extraction of 4 factors, which explained 55.6% of the total variance. Factor 1, communication, explained 17.8% of the total variance; factor 2, expression of emotions, explained 13.4% of the total variance; factor 3, problem-solving, explained 12.9% of the total variance; factor 4, cooperation, explained 11.4% of the total variance. Furthermore, the scree plot chart supported the retention of the four factors, based on the inflection point of the curve. Slightly changes have emerged from the EFA, in relation to the original scale: the order of the factors was changed; the factor “collaboration and problem-solving,” was split into two different factors, “problem-solving” and “cooperation”; and the factor “behavior” was removed, since the items of this factor have saturated in the remaining factors. Items 14, 15, and 17 were moved from factor 4 “behavior” to factor 1 “communication,” item 5 was moved from factor 2 “collaboration and problem-solving” to factor 2 “expression of emotions,” and items 4 and 16 were moved from factor 1 “expressing emotions” and from factor 4 “behavior,” respectively, to factor 3 “problem-solving.” The proportion of variance for each variable explained by the factors, which is usually referred to as communality (*h*^2^), was >.40 reference value (Marôco, 2021b) for all communalities, except for item 8.

**Table 3.** Portuguese Version of Iceland-Expressive Family Functioning Questionnaire Factorial Structure.

Items	Factor 1	Factor 2	Factor 3	Factor 4	*h* ^2^
1. I know when my family members are expressing their feelings, for example, joy, anger, and sadness.		.660			.448
2. I know how my family members react when one of us is sad.		.816			.726
3. I know how my family members react when I say what I think.		.598			.513
4. I know who most of my family would go to if they needed to talk to someone.			.636		.492
5. I notice the change in family relations when problems have been solved.		.531			.576
6. If my family and I have other problems in our lives, I know how we will deal with them.			.682		.609
7. I know the effect it has on my family members when we help each other with everyday domestic chores.				.793	.706
8. I know who would be the first to realize whether the family members were working as a team instead of competing against each other.				.463	.369
9. I know how family members feel when everyone is responsible for the daily household chores.				.677	.569
10. Family members talk about their feelings.	.578				.545
11. Family members express themselves clearly and honestly when talking to each other.	.666				.490
12. Family members find ways to have honest and useful conversations (e.g., face-to-face, by telephone, or by e-mail).	.667				.533
13. Every family member knows when problems arise.	.712				.565
14. I know what each and every family member does when one of us is annoyed.	.509				.554
15. I know the reaction of all members of the family when they speak honestly with each other.	.549				.604
16. I know what each family member does in difficult situations.			.726		.672
17. I know how the members of the family react when relating to each other (this means behavior such as slamming doors, not talking, offering something to eat, and giving it time).	.496				.477
Factors	Eigen value		% Variance	% Cumulative variance	
Factor 1—Communication	5.441		17.801	17.801	
Factor 2—Expression of emotions	1.590		13.428	31.229	
Factor 3—Problem-Solving	1.277		12.932	44.161	
Factor 4—Cooperation	1.140		11.420	55.580	

*Note. h*^2^ = Communalities.

#### Confirmatory Factor Analysis (CFA)

The four-factor solution of the questionnaire was assessed utilizing CFA (Marôco, 2021a). In the assessment of normality, the items showed response heterogeneity, with minimum and maximum indices ranging from 1 to 5. We assessed the sensitivity of each item, using asymmetry (*Sk* ≤ 3) and flattening (*Ku* ≤ 7). Results revealed asymmetry values oscillating in absolute values, between –1.45 and –0.18, flattening values between –1.09 and 1.97, and a multivariate Mardia coefficient of 4.62 (*Cm* ≤ 5), which indicate a normal distribution (Marôco, 2021a). The critical ratios, or *z* values, were all statistically significant (*p* < .001), which led to the retention of all the items.

**Figure 2.**
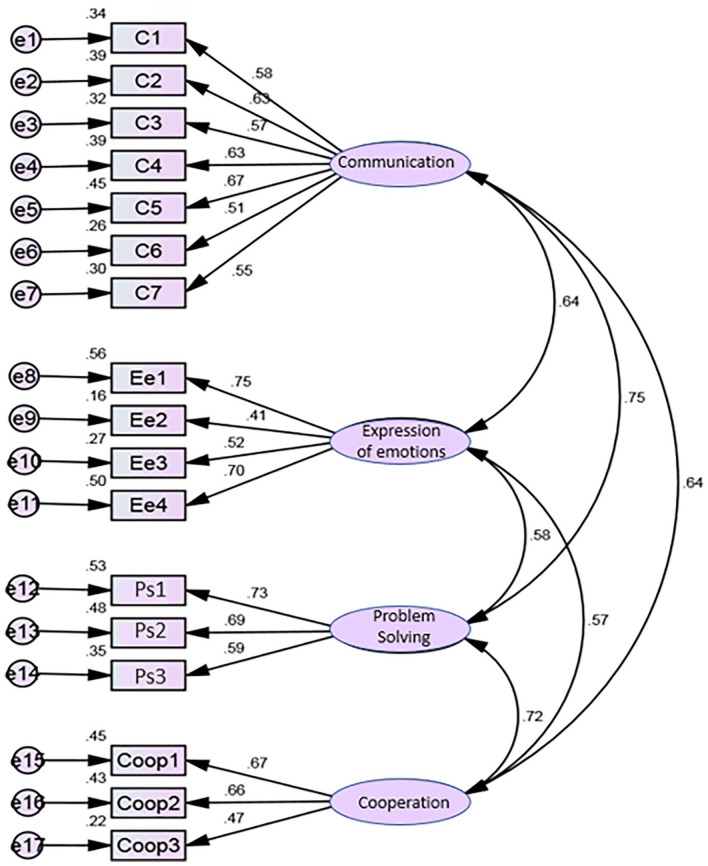
Portuguese version of ICE-EFFQ confirmatory factor analysis—initial model. *Note.* ICE-EFFQ = Iceland-Expressive Family Functioning Questionnaire.

As shown in Figure 2, the trajectories of the items with the factors to which they correspond had high factor weights (*λ* ≥ .50), except for item 1 (emotions 2 [Ee2 *λ* = .41]) and item 8 (cooperation 3 [Coop3 *λ* = .47]). The reference values may, in exploratory studies, be between .40 and .50, so it was not necessary to eliminate any items, and CFA could proceed. Individual reliability was adequate (*r*^2^ ≥ .25) in the four subscales, except for the cooperation subscale in relation to item Coop3 (*r*^2^ = .22). As shown in Table 4, in the initial model, the global goodness-of-fit indices showed a good fit with χ^2^/*gl*., CFI, RMSR, and SRMR and acceptable fit with GFI and RMSEA. We then proceeded to refine the model, based on the modification index indicated by AMOS, which correlated errors 2 (commun11) and 3 (commun12). There were no problems of multicollinearity, that is, there were no correlations between items, revealing that the items were independent. We note that, after model refinement, the global fit remained unchanged for all global fit indices (see Table 4).

**Table 4.** Portuguese Version of Iceland-Expressive Family Functioning Questionnaire Global Adjustment Ratios.

Model	χ^2^/*df*	GFI	CFI	RMSEA	RMSR	SRMR
Initial model (Figure 2)	1.444	.872	.904	.061	.087	.071
Refined model	1.384	.878	.917	.057	.085	.069
Second-order model (Figure 3)	1.426	.871	.906	.060	.087	.071

*Note.* χ^2^/*df* = Chi-square statistic ratio/degrees of freedom; GFI = goodness-of-fit index; CFI = comparative fit index; RMSEA = root mean square error of approximation; RMSR = root mean square residual; SRMR = standardized root mean square residual.

**Figure 3.**
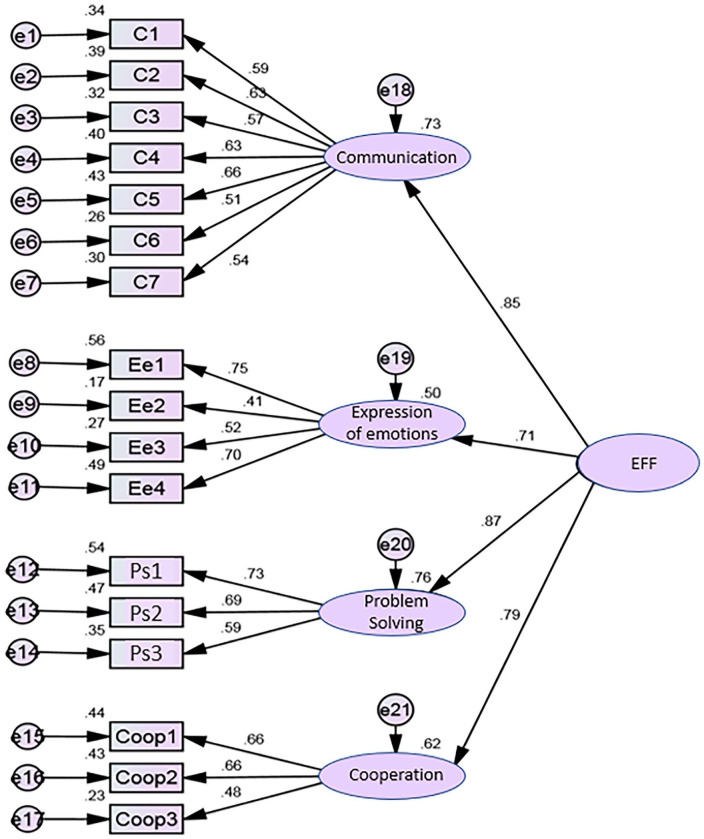
Portuguese version of ICE-EFFQ confirmatory factor analysis—second-order model. *Note.* ICE-EFFQ = Iceland-Expressive Family Functioning Questionnaire; EFF = expressive family functioning.

Given that the high correlational values between factors were suggestive of the existence of a second-order factor, we proposed a hierarchical structure with a second-order factor, which we designated as expressive family functioning (EFF). Figure 3 illustrates the model obtained. Analysis shows the following: factor 1—communication explained 73% of global factor 5—EFF; Factor 2—expression of emotions explained 50% of global factor 5—EFF; factor 3—problem-solving explained 76% of global factor 5—EFF; and factor 4—cooperation explained 62% of global factor 5—EFF. The lowest correlation registered with the global factor was observed in factor 2 (*r* = .71) and the highest with factor 3 (*r* = .87). As can be observed in Table 4, the global fit indices remained unchanged in the second-order model, compared with those recorded in the initial model and the refined model.

Table 5 represents CR, AVE, and DV. Factors 1 and 3 had adequate (>.70) internal consistency (CR) and factors 2 and 4 had poor (<.70). The AVE showed that all four factors had a lower value than recommended (≥.50). There was no convergent validity among all the factors. The DV was only evident between factor 2 and factor 3 and between factor 2 and factor 4 since the squared correlational values were lower than the AVE. In addition, the stratified coefficient was high (.91), with .38 AVE (Marôco, 2021a). Table 6 represents the convergent/divergent validity of items. All items had convergent validity with the corresponding factor since the correlational value was higher with the subscale to which the item belonged, followed by the correlational value of the item with the total factor of the scale.

**Table 5.** Portuguese Version of Iceland-Expressive Family Functioning Questionnaire Composite Reliability, Average Variance Extracted, and Discriminant Validity.

Factors	CR	AVE	Discriminant validity		
			F2	F3	F4
F1—Communication	.788	.349	.409	.562	.409
F2—Expression of emotions	.692	.426		.336	.324
F3—Problem-solving	.710	.476			.518
F4—Cooperation	.628	.365			

*Note.* CR = composite reliability; AVE = average variance extracted.

**Table 6.** Items Convergent /Divergent Validity.

Items	F1	F2	F3	F4	Ftotal
1. I know when my family members are expressing their feelings, for example, joy, anger, and sadness.	.149	.659**	.119	.136	.332**
2. I know how my family members react when one of us is sad.	.343**	.817**	.282*	.357**	.567**
3. I know how my family members react when I say what I think.	.214*	.682**	.320**	.258*	.451**
4. I know who most of my family would go to if they needed to talk to someone.	.348**	.280*	.786**	.347**	.541**
5. I notice the change in family relations when problems have been solved.	.537**	.706**	.433**	.352**	.669**
6. If my family and I have other problems in our lives, I know how we will deal with them.	.453**	.289**	.807**	.408**	.614**
7. I know the effect it has on my family members when we help each other with everyday domestic chores.	.354**	.251*	.431**	.755**	.530**
8. I know who would be the first to realize whether the family members were working as a team instead of competing against each other.	.392**	.293**	.287**	.678**	.506**
9. I know how family members feel when everyone is responsible for the daily household chores.	.354**	.311**	.369**	.809**	.543**
10. Family members talk about their feelings.	.719**	.221*	.410**	.410**	.621**
11. Family members express themselves clearly and honestly when talking to each other.	.674**	.209*	.277**	.334**	.543**
12. Family members find ways to have honest and useful conversations (e.g., face-to-face, by telephone, or by e-mail).	.715**	.271*	.408**	.291**	.608**
13. Every family member knows when problems arise.	.678**	.201*	.370**	.258*	.551**
14. I know what each and every family member does when one of us is annoyed.	.581**	.391**	.228*	.303**	.532**
15. I know the reaction of all members of the family when they speak honestly with each other.	.674**	.446**	.516**	.290**	.667**
16. I know what each family member does in difficult situations.	.492**	.396**	.797**	.394**	.660**
17. I know how the members of the family react when relating to each other (this means behavior such as slamming doors, not talking, offering something to eat, giving it time).	.606**	.357**	.279**	.386**	.565**

*Note.* ^**^p <.001 ^*^p <.005.

Bold faced values = items correlational value with the subscale to which the item belong.

### Reliability Assessment

The analysis of the scale’s reliability, as shown in Table 7, revealed that the mean indices and their standard deviations were all above the midpoint, oscillating from 3.17 ± 1.35 in item 10 to 4.29 ± 0.92 in item 7. In the correlation coefficients, corrected item-total, all items had correlations >.20 reference value (Pestana & Gageiro, 2014), so none were excluded. The item that presented the lowest stability (*r* = .23) was item 1. The one with the maximum correlation (*r* = .60) was item 15. Cronbach’s alpha for each item was ≥.85, with a global alpha of .86. The test–retest correlation showed values of temporal stability for the global scale of *r* = .75 (*p* < .001), and for the subscales: communication *r* = .66 (*p* < .001); expression of emotions *r* = .55 (*p* < .001); problem-solving *r* = .66 (*p* < .001); and cooperation *r* = .64 (*p* < .001). The calculation of the ICC indicated, as shown in Table 8, that the precision of the instrument’s estimates was highly significant (*p* < .001), for the total scale and for the four subscales, and that the values for temporal stability were all satisfactory in the interval estimate of 95% confidence (A. C. de Souza et al., 2017; Polit & Beck, 2017).

**Table 7.** Portuguese Version of Iceland-Expressive Family Functioning Questionnaire Internal Consistency.

Items	*M*	*SD*	Item/total correlation *r*/item total	Squared multiple correlation *R*^2^	α
EEmo1					
I know when my family members are expressing their feelings, for example, joy, anger, and sadness.	3.98	1.165	.229	.222	.866
EEmo2					
I know how my family members react when one of us is sad.	3.98	1.037	.495	.499	.854
EEmo3					
I know how my family members react when I say what I think.	3.88	1.077	.365	.306	.859
EEmo4					
I know who most of my family would go to if they needed to talk to someone.	3.79	1.204	.453	.313	.856
ColProbSolv5					
I notice the change in family relations when problems have been solved.	3.87	1.161	.602	.474	.848
ColProbSolv6					
If my family and I have other problems in our lives, I know how we will deal with them.	3.36	1.182	.537	.412	.852
ColProbSolv7					
I know the effect it has on my family members when we help each other with everyday domestic chores.	4.29	.917	.463	.393	.855
ColProbSolv8					
I know who would be the first to realize whether the family members were working as a team instead of competing against each other.	4.08	1.005	.430	.234	.856
ColProbSolv9					
I know how family members feel when everyone is responsible for the daily household chores.	3.91	1.162	.459	.353	.855
Commun10					
Family members talk about their feelings.	3.17	1.352	.533	.452	.852
Commun11					
Family members express themselves clearly and honestly when talking to each other.	3.81	1.171	.458	.379	.855
Commun12					
Family members find ways to have honest and useful conversations (e.g., face-to-face, by telephone, or by e-mail).	3.83	1.172	.531	.447	.852
Commun13					
Every family member knows when problems arise.	3.81	1.164	.467	.384	.855
Behavior14					
I know what each and every family member does when one of us is annoyed.	4.10	.898	.467	.387	.855
Behavior15					
I know the reaction of all members of the family when they speak honestly with each other.	3.90	1.099	.604	.506	.849
Behavior16					
I know what each family member does in difficult situations.	3.49	1.156	.591	.531	.849
Behavior17					
I know how the members of the family react when relating to each other (this means behavior such as slamming doors, not talking, offering something to eat, and giving it time).	4.00	1.017	.495	.357	.854
Global Cronbach’s alpha coefficient			0.863		
Test–retest correlation					
Global scale			.750		
Communication subscale			.661		
Expression of emotions subscale			.553		
Problem-solving subscale			.655		
Cooperation subscale			.641		

*Note. r* = Pearson correlation coefficient; *R*^2^ = determination coefficient; α = Cronbach’s alpha coefficient.

**Table 8.** Intraclass Correlation Coefficient (95% CI) for the Test–Retest Group (*N* = 40).

Intraclass correlation coefficient (95% CI)					
Scale	ICC^b^	Confidence interval 95%		*p* value	α^c^
		Lower bound	Upper bound		
Global scale	.732^a^	.548	.849	.000	.845
Subscale 1—Communication	.661^a^	.443	.805	.000	.796
Subscale 2—Expression of emotions	.546^a^	.286	.731	.000	.707
Subscale 3—Problem-solving	.650^a^	.427	.798	.000	.788
Subscale 4—Cooperation	.599^a^	.356	.766	.000	.749

*Note.* CI = confidence interval; ICC = intraclass correlation. Two-way mixed effects model where people effects are random and measures effects are fixed.

a The estimator is the same whether the interaction effect is present or not.

b Enter intraclass correlation coefficients C using a consistency definition. The between-measure variation is excluded from the denominator variation.

c This estimate is calculated assuming that the interaction effect is absent because it is not estimable otherwise.

Based on the final version of the scale, as seen in Table 9, we finished studying the scale, referring to the mean of the global scale and the study of internal consistency by subscales of the remaining items. The mean scores for all the items, for the four factors and for the global scale, were all above the midpoint.

**Table 9.** Portuguese Version of ICE-EFFQ Subscales Internal Consistency Reliability.

Items	Subscales	*M*	*SD*	*r*/item total	Squared multiple correlation *R*^2^	α if item deleted
	Communication	26.63	5.265		α .789	
10	Family members talk about their feelings.	3.17	1.352	.553	.341	.756
11	Family members express themselves clearly and honestly when talking to each other.	3.81	1.171	.521	.316	.761
12	Family members find ways to have honest and useful conversations (e.g., face-to-face, by telephone, or by e-mail).	3.83	1.172	.577	.399	.750
13	Every family member knows when problems arise.	3.81	1.164	.528	.302	.760
14	I know what each and every family member does when one of us is annoyed.	4.10	.898	.450	.264	.775
15	I know the reaction of all members of the family when they speak honestly with each other.	3.9	1.099	.533	.301	.759
17	I know how the members of the family react when relating to each other (this means behavior such as slamming doors, not talking, offering something to eat, and giving it time).	4	1.017	.460	.250	.772
	Expression of emotions	15.69	3.17		α .684	
1	I know when my family members are expressing their feelings, for example, joy, anger, and sadness.	3.98	1.165	.361	.170	.678
2	I know how my family members react when one of us is sad.	3.98	1.037	.647	.424	.492
3	I know how my family members react when I say what I think.	3.88	1.077	.424	.213	.634
5	I notice the change in family relations when problems have been solved.	3.87	1.161	.433	.264	.630
	Problem-solving	10.64	2.82		α .711	
4	I know who most of my family would go to if they needed to talk to someone.	3.79	1.204	.502	.252	.656
6	If my family and I have other problems in our lives, I know how we will deal with them.	3.36	1.182	.548	.303	.598
16	I know what each family member does in difficult situations.	3.49	1.156	.539	.294	.610
	Cooperation	12.28	2.314		α .608	
7	I know the effect it has on my family members when we help each other with everyday domestic chores.	4.29	.917	.480	.251	.422
8	I know who would be the first to realize whether the family members were working as a team instead of competing against each other.	4.08	1.005	.315	.100	.635
9	I know how family members feel when everyone is responsible for the daily household chores.	3.91	1.162	.463	.250	.427
	Total QFEF	65.24	10.614		α .863	

*Note.* ICE-EFFQ = Iceland-Expressive Family Functioning Questionnaire; *r* = Pearson correlation coefficient; *R*^2^ = coefficient of determination; α = Cronbach’s alpha; QFEF= Questionário de Funcionamento Expressivo da Família Portuguese version of ICE-EFFQ.

In factor 1—communication, the mean values showed homogeneity in the responses given to the different items, since the scores obtained range from 3.17 ± 1.35 in item 10 to 4.10 ± 0.90 in item 14. Cronbach’s alpha coefficients per item indicated reasonable internal consistency if the item is eliminated; the lowest value (*α* = .75) is found for item 12 and the highest (*α* = .78) for item 14. The internal consistency obtained for the communication subscale was also reasonable (*α* = .79). Item 12 was the one that correlated the most with communication (*r* = .58) with a variability of 39.9%, instead of item 14 (*r* = .45), which correlates the least with factor 1 with a percentage of explained variance of 26.4%.

Regarding factor 2—expression of emotions, the mean values showed homogeneity in the responses given to the different items, since the scores ranged from 3.87 ± 1.16 in item 5 to 3.98 (±1.17 in item 1 and ±1.04 in item 2). Cronbach’s alpha revealed weak internal consistency for items 1 (*α* = .68), 3 (*α* = .63), and 5 (*α* = .63); unacceptable for item 2 (*α* = .49); and weak for the subscale (*α* = .68). The item that correlated the most with the overall results of factor 2 was item 2 (*r* = .65) and the lowest correlation was for item 1 (*r* = .36) with percentages of explained variance of 42.4% and 17%, respectively.

In the result analysis of factor 3—problem-solving, the mean indices oscillated between 3.36 ± 1.18 in item 6 and 3.79 ± 1.20 in item 4. Cronbach’s alpha indicated poor internal consistency for the items; the lowest value (*α* = .60) was for item 6 and the highest (*α* = .66) for item 4. The subscale featured a total alpha of .71, indicative of reasonable internal consistency. Item 6 was the one that correlated the most with problem-solving (*r* = .55) with a variability of 30.3%, instead of item 4 (*r* = .50), which correlated the least with factor 3 with a percentage of explained variance of 25.2%.

Regarding factor 4—cooperation, homogeneity was observed in the responses, with mean values ranging between 3.91 ± 1.16 for item 9 and 4.29 ± 0.92 for item 7. Cronbach’s alpha indicated unacceptable internal consistency for items 7 (*α* = .42) and 9 (*α* = .43) and weak for item 8 (*α* = .64) and for the subscale (*α* = .61). The item that correlated the most with the overall results of factor 4 was item 7 (*r* = .48) and the one with the lowest correlation was item 8 (*r* = .32), with percentages of explained variance of 25.1% and 10%, respectively.

Factor analysis of the scale was then completed by presenting the Pearson correlation matrix between the several factors and the global scale. As shown in Table 10, the correlations between the different subscales showed moderate to high correlation values with the total factor of the scale, explaining 49.3% to 76.7% of the total variance. The correlation matrix between the four factors and the global scale revealed that the correlations were positive and significant (*p <* .001), indicating that an increase or decrease in the indices of a variable corresponded to an increase or decrease of the variable with which it correlated. The lowest correlational value between subscales was between factor 4 and factor 2 (*r* = .38), with an explained variance percentage of 14.7%, and the highest correlational value was between factor 3 and factor 1 (*r* = .54), with an explained variance of 29.2%.

**Table 10.** Portuguese Version of ICE-EFFQ Factors’ Pearson Correlation Matrix.

Factors	F1	F2	F3	F4
Communication—F1	—			
Expression of emotions—F2	.437**	—		
Problem-solving—F3	.540**	.403**	—	
Cooperation—F4	.488**	.383**	.481**	—
Global Factor	.876**	.706**	.759**	.702**

*Note.* ICE-EFFQ = Iceland-Expressive Family Functioning Questionnaire; F1 = communication; F2 = expression of emotions; F3 = problem-solving; F4 = cooperation.

** *p* < .001.

## Discussion

Nurses who work with families dealing with acute or chronic illnesses need to know the effects of their interventions on families. Valid and reliable instruments that can measure family functioning, including expressive functioning, therapeutic change, and the results of nursing interventions on families, are needed in both clinical and research contexts (Chesla, 2010; Gisladottir & Svavarsdottir, 2016; Sveinbjarnardottir et al., 2012). This study aimed to adapt the ICE-EFFQ (Sveinbjarnardottir et al., 2012), for European Portuguese and assess the psychometric properties of the Portuguese version “Questionário de Funcionamento Expressivo da Família (QFEF)” (Questionnaire on the Expressive Family Functioning [QEFF]). To the best of our knowledge, this is the first valid and reliable instrument in the Portuguese context, designed to measure expressive family functioning in families affected by a member’s acute or chronic mental illness.

Regarding the translation and adaption process, the use of a rigorous and systematic method developed in five steps (Sousa & Rojjanasrirat, 2011), with the involvement of expert translators and the use of an expert panel from distinct areas of nursing (family nursing, mental health and psychiatric nursing, and community nursing), ensured the content equivalence of the instrument, improved the adaptation of the instrument to the Portuguese context, and provided the content validity of the Portuguese version. An excellent agreement among experts on items’ relevance for measuring the construct and optimal values for CVI at the item level and at the scale level (Polit & Beck, 2006, 2017; Streiner et al., 2015) was achieved, evidencing a strong relevance of the items in the Portuguese version. The pre-testing strengthened the conceptual, semantic, and content equivalency (Polit & Beck, 2004, 2017) of the pre-final version of the translated instrument, ensuring the QFEF’s face validity and generating the final European Portuguese version of the ICE-EFFQ, for psychometric testing.

The results of psychometric testing were obtained from 54 (44.6%) patients and 67 (55.4%) family members, mostly female (66.1%), with a minimum age of 18 years and a maximum of 75 years, and an average age of 44.9 years (*SD* = 14.5) for the total sample. In comparison with the Icelandic (Sveinbjarnardottir et al., 2012) and Danish (Konradsen et al., 2018) studies, there was a predominance of females and similar mean age, except for the Danish sample, where the mean age (61; *SD* = 14.1) was much higher. Family members related to the patient, as well as in the Icelandic original study (Sveinbjarnardottir et al., 2012), were mostly sons/daughters, spouses, and parents (85%), reinforcing the strong Portuguese tradition of the closest relatives being caregivers of sick family members.

The distribution of responses on family functioning in this study revealed high scores, which are considered to represent good family functioning. The mean indices and their standard deviations were well centered, all being above the midpoint of the rating scale, meaning that, on average, families were functioning well in all domains of family functioning. Although no score in the Portuguese version suggests optimal family functioning, the cooperation subscale was the one that scored the highest, pointing to a very good family functioning, in this dimension of family functioning. The overall high scores may indicate that Madeiran families function well even when they must deal with an acute or chronic family illness, such as depression. Comparable results were found in the Danish study (Konradsen et al., 2018), despite the differences between the therapeutic settings and the Danish and Portuguese cultures. Our results are not in line with those of Daches et al. (2018), Pérez et al. (2018), and Sell et al. (2021), who reported an impaired family functioning, in families with a member with mental illness. This divergence of results may be related to differences in culture, sample composition, perception bias (de Los Reyes et al., 2008; Haack et al., 2017; Sell et al., 2021), or the use of different assessment tools. Furthermore, as stated by Sell et al. (2021), Portuguese family members may have tended to overestimate family functioning and conceal family problems, to protect the member with mental illness.

The final factor solution derived from the EFA, with all factorial loads above .4 (Marôco, 2021b), confirmed the 4-factor structure of the original instrument (Sveinbjarnardottir et al., 2012), with a lower explained variance (55.6%) than the original scale (60.3%). In this factor solution, six items were found to load onto factors other than those on which they had saturated on the original scale (ICE-EFFQ), resulting in the splitting of the “problem-solving and collaboration” factor into two factors, in the removal of the “behavior” factor, and in the Portuguese version being a modified version of the ICE-EFFQ. Such differences may be due to the following reasons: First, cultural characteristics of Icelandic and Portuguese populations, may have influenced the response of the participants to the questionnaire, in each context. The authors of the original instrument (Sveinbjarnardottir et al., 2012) and the authors of the Danish study (Konradsen et al., 2018) addressed this issue, pointing out that the cultural context might influence family functioning. Second, in the Icelandic study, the sample consisted of family members, while, in the Portuguese study, as proposed by Chesla (2010), the sample was made up of patients and family members. As stated in the literature, the validity of an instrument should be thought of as a characteristic of the instrument itself when applied to a sample. That is, the structure of a scale can be directly influenced by the characteristics of the population under study. Third, the ICE-EFFQ (Sveinbjarnardottir et al., 2012) was tested on family members of patients with various kinds of diseases (medical, surgical, pediatric, geriatric, and psychiatric), while the QFEF was assessed in a more restricted population of patients with depression and their family members. We believe that the cognitive and functional losses and emotional changes associated with depression (de Almeida, 2018; World Health Organization, 2017, 2021) may have influenced the interpretation and response of depressed individuals to the questionnaire (Daches et al., 2018; Pérez et al., 2018; Sell et al., 2021). Furthermore, although the sample composition was 44.6% of patients, we found that 50.4% of the participants had depression, and 56.2% had a mental illness. The presence of such health condition, usually associated with abnormal thoughts, perceptions, emotions, behaviors, and relationships with others (World Health Organization, 2019), in such a high percentage of participants, may have influenced the results of the Portuguese questionnaire, justifying the differences found in comparison with the Icelandic instrument (Sveinbjarnardottir et al., 2012). In addition, there are concerns on the support to be provided to these families, since we found that, beyond patients, 20.9% of the family members were suffering from a psychiatric illness. It is suggested that more studies with larger samples including patients and family members should be conducted in the clinical context of depression. Correlational studies should also be done, to clarify to what extent the presence of mental illness may influence the scale results.

All items of the QFEF presented a good sensitivity, with an acceptable range of asymmetry (*Sk* < 3) and flattening (*Ku* < 7) values (Marôco, 2021a). All items had high factor weights (*λ* ≥ .50) with the factors to which they corresponded, except items 1 and 8. Items with saturations lower than .50, in a more conservative analysis, should be eliminated. However, the decision was made to keep them because the study was preliminary. Furthermore, a factor must have at least 3 items, and if this rule were followed, and item 8 was eliminated from factor 4, this factor would also be eliminated. Individual reliability was also adequate (*r*^2^ ≥ .25) in the four subscales, showing the relevance of the factors to predict the items.

CFA has shown acceptable (GFI and RMSEA) to good (χ^2^/*gl*., CFI, RMSR, and SRMR) values, in the goodness of fit indices. The RMSEA is a measure of the amount of error in the CFA that should be minimal (Marôco, 2021a). Values <.05 show a good fit between the proposed model and the observed matrix, while values <.08, indicate an acceptable fit (Marôco, 2021a; Pestana & Gageiro, 2014). The RMSEA was found to have a perfect fit (.00) in the ICE-EFFQ (Sveinbjarnardottir et al., 2012) and a poor fit (.11) in the Danish version (Konradsen et al., 2018). The differences between our results and those from the Icelandic and Danish instruments might be explained by differences in sample composition and cultural and clinical settings. The GFI showed an acceptable fit (.87), as well as in the Danish version (.80). Such results might be related to the small sample size, since GFI tends to increase, with an increase in sample size and the number of model variables (Marôco, 2021a, 2021b; Pestana & Gageiro, 2014; Soeken, 2010). Further studies with a larger sample size are recommended for greater sensitivity. All fit indices showed values within the cutoff point, indicating that the four-factor model fits the data and that there is construct validity.

The analysis of CR showed indices >.9 for the total scale and a range between .628 and .788, for the four domains. The CR estimates the internal consistency of items relative to the factor, indicating the degree to which these items are consistently manifestations of the latent factor. A suitable construct reliability has a cutoff point of .7, although lower values are acceptable for exploratory investigations (Marôco, 2021a). It follows that, according to the principles of internal consistency, the Portuguese version of the ICE-EFFQ exhibits measurement reliability.

The convergent validity of the 4 factors estimated by AVE, (AVE F1=.35; AVE F2=.43; AVE F3=.48; AVE F4=.37) was lower than the reference values (≥.50). Therefore, there was no convergent validity among all factors. There was evidence of DV between factor 2 (expression of emotions) vs. factor 3 (problem-solving), and between factor 2 (expression of emotions) vs. factor 4 (cooperation), since the squared correlational values between the factors were lower than AVE, meaning that these factors measure different facets of family expressive functioning. It should be noted that, for the global scale, the stratified coefficient was high (.91), with .38 AVE. Based on these results, the instrument is appropriate for this sample, so it may be a valuable resource for the study of family expressive functioning in the Portuguese population. The adjustment of the 4-factor model was acceptable, with factor weights greater than the reference value (.40), and adjustment quality indices supporting the 4 dimensions structure in the modified Portuguese version: factor 1—communication (7 items); factor 2—expression of emotions (4 items); factor 3—problem-solving (3 items); and factor 4—cooperation (3 items).

The QFEF reliability achieved a good internal consistency for the global scale (*α* = .86), and was acceptable for all four factors (Pestana & Gageiro, 2014). Compared with the ICE-EFFQ (Sveinbjarnardottir et al., 2012) and the Danish version of the instrument (Konradsen et al., 2018), the QFEF presented lower values for Cronbach’s alpha for the total scale and for the four subscales. This may be related to the small sample size, or to cultural differences as suggested by the authors of the original questionnaire (Sveinbjarnardottir et al., 2012). In that sense, more testing with larger samples and among participants from distinct cultural backgrounds and with different family illnesses is required, since cross-cultural studies are essential to strengthening the psychometric properties of a scale (Alfaro-Díaz et al., 2020; Rodrigues et al., 2021). The test–retest correlation displayed good values of temporal stability for the global scale, and satisfactory values for all subscales. Test–retest reliability analysis showed that the instrument is stable over time, meaning that it can yield a similar score when administered under the same conditions, to the same participants, and at separate times. The internal consistency and test–retest reliability showed good reliability of the QFEF in the context of its application, supporting the instrument’s construct validity and confirming that it is a reliable measure (Polit & Yang, 2015). The QFEF presents adequate factor validity, sensitivity, and reliability, is available in Portuguese and English, and has the potential to measure family expressive functioning, before and after family nursing interventions, when family members face an acute or chronic mental illness of a close relative. The results of this study ensure the validity and reliability of the Portuguese version of the ICE-EFFQ, warranting its usefulness and suitability for Portuguese health care settings.

### Limitations

One limitation of this study might be the small sample size and the non-randomization of the sample, associated with difficulties in selecting participants. A selection bias could occur, since participants were intentionally selected, although according to the inclusion and exclusion criteria. Considering that the questionnaire has been psychometrically tested in a specific population of depressed patients and their family members, the validity and reliability of the Portuguese version of the ICE-EFFQ were achieved for the sample under study and cannot be extrapolated to different samples or clinical settings. Further empirical testing correlating the QFEF with other measures could also have strengthened the validity and reliability of the instrument. It is worth noting that correlations between males and females, patients, and family members, depressed and non-depressed participants, and between older and younger than 50 years of age were not assessed. Therefore, it will be of interest that, in future studies, the evaluation of these correlations will be considered.

### Strengths

Regarding the identified strengths, the fact is highlighted that the QFEF is the first sensitive, valid, and reliable instrument available in European Portuguese, to assess expressive family functioning in the context of its application. It derives from the ICE-EFFQ (Sveinbjarnardottir et al., 2012), an Icelandic questionnaire grounded on a conceptual framework of family nursing, the CFAM (Wright & Leahey, 2013), which has deep-rooted theoretical foundations on many years of clinical experience with families facing acute or chronic illnesses of their relatives. Furthermore, although there are instruments for assessing family functioning that have been psychometrically tested and have been applied by nurses, they focus on family functioning in healthy families and are based on conceptual frameworks from scientific areas other than nursing (sociology and other health sciences).

The QFEF has a good reproducibility, and there is a great deal of strength on its ability to assess family functioning, before and after family nursing interventions. It is a particularly useful and easy-to-apply instrument, which measures the therapeutic change and the outcomes of nursing interventions on families. This questionnaire is not intended to determine whether families are emotionally healthy or not and does not classify families as functional or dysfunctional, although it assesses expressive family functioning and the dimensions considered essential for a healthy family functioning, The QFEF fills in a gap in the availability of instruments to assess family functioning in Portugal and will be valuable in the clinical activity of nurses specializing in mental health nursing and psychiatry, and of all nurses whose professional practice involve family intervention, regardless of the context.

## Conclusion

Family functioning is a concept that has been widely studied in social science research, health care, and nursing sciences. Valid and reliable instruments from the areas of sociology and health sciences have been applied by nurses to assess family functioning in healthy families. However, to respond to the needs of families faced with illness experiences, it is essential that, in the clinical context, there be valid and reliable instruments that assess family functioning, therapeutic change, and the effect of nursing interventions on families (Chesla, 2010; Mattila et al., 2009; Sveinbjarnardottir et al., 2012). The QFEF, the European Portuguese version of the ICE-EFFQ (Sveinbjarnardottir et al., 2012), is a sensitive, valid, and reliable instrument, available in Portuguese and English, to assess expressive family functioning in families facing mental illness of their members. It was rigorously translated, culturally adapted, and psychometrically assessed with robust statistical tests, which confirmed its validity and reliability in the context of Portuguese families with depressed members. Content validity is well established exhibiting an excellent agreement among experts and optimal values for CVI. EFA confirmed the original four-factor structure (Sveinbjarnardottir et al., 2012), although with slight differences in item structure, which resulted in the Portuguese version of the ICE-EFFQ being a modified version of the original instrument. CFA showed that there is construct validity, with acceptable to good values of goodness of fit indices (Marôco, 2021a). Internal consistency and test–retest reliability also showed good reliability of the QFEF.

The QFEF is an easy-to-apply self-report questionnaire, takes approximately 10 minutes to complete, and can be applied in psychiatric hospitals and community health centers. This is a useful instrument for nurse researchers, educators, managers, practice nurses, and other health professionals working with families facing mental illness of their members, to measure family functioning, evaluate therapeutic change, assess the outcomes of family interventions, improve nursing practice with families, and foster the nurses’ spirit of scientific curiosity, contributing to supporting the emerging translational research. The QFEF is a powerful therapeutic and research instrument, with a large potential of application in a wide range of clinical settings. We strongly believe that the QFEF is a valuable instrument for clinical practice, as it provides a standardization of procedures and a methodological working guideline, for the mental health professionals who intervene with families dealing with a member’s mental illness. The practical applicability of this instrument will add value to the professional performance of health care professionals, working in this area. It will also contribute to the health indicators production (useful for management, research, education, and development), promoting the assurance of continuous quality improvement in mental health care provided to families.

With the application of this instrument, it is possible to know how families are functioning at a given time and to intervene in the overall family functioning as well as in its most vulnerable components. The QFEF will help to tailor interventions, according to each family’s specific needs, with the purpose of softening family suffering and improve, promote, and/or maintain a good family functioning as well as the family mental health. Altogether, it enhances the visibility on mental health nurses’ therapeutic role in their intervention with families. Further studies with the QFEF are suggested, with larger samples, greater diversity of family health problems, and in different clinical settings, to ensure and strengthen the validity and reliability of the instrument and expand its use. Therefore, the cultural adaptation and validation of this instrument into European Portuguese leaves open for nurses and other health professionals the possibility and scientific curiosity of its application, validation, and dissemination in other clinical and cultural contexts. The applicability of this instrument in families with adult members with depression, and in other family illness contexts, may constitute an added value for better family mental health and for better general family health.
